# Carbon Monoxide-Releasing Activity of Plant Flavonoids

**DOI:** 10.1021/acs.jafc.4c09069

**Published:** 2024-12-31

**Authors:** Lucie Muchová, Mária Šranková, Sriram Balasubramani, Panshul Mehta, Dafni Vlachopoulou, Akshat Kapoor, Andrea Ramundo, Yann Anton Jézéquel, Igor Bożek, Martina Hurtová, Petr Klán, Vladimír Křen, Libor Vítek

**Affiliations:** a Institute of Medical Biochemistry and Laboratory Diagnostics, and 4th Department of Internal Medicine, General University Hospital in Prague and 1st Faculty of Medicine, Charles University, Na Bojišti 3, Prague 2 12108, Czech Republic; b Department of Chemistry, Faculty of Science, Masaryk University, Kamenice 5, Brno 62500, Czech Republic; c RECETOX, Faculty of Science, Masaryk University, Kamenice 5, Brno 62500, Czech Republic; d Laboratory of Biotransformation, Institute of Microbiology of the Czech Academy of Sciences, Vídeňská 1083, Prague 4 CZ 14200, Czech Republic

**Keywords:** quercetin, 2,3-dehydrosilybin, carbon monoxide, photoinduced release, oxidative stress, mitochondrial respiration, cell cycle, photoCORM

## Abstract

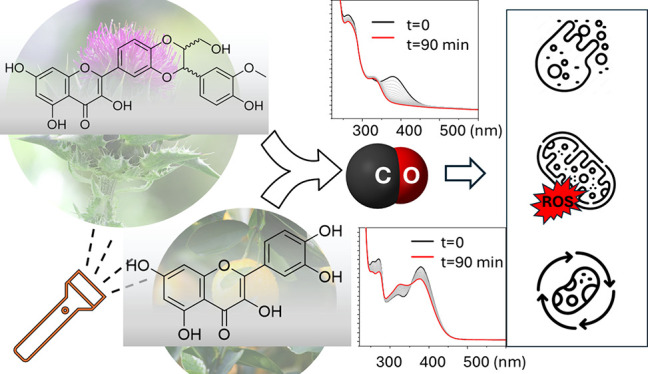

Flavonoids are naturally occurring compounds found in fruits, vegetables, and other plant-based foods, and they are known for their health benefits, such as UV protection, antioxidant, anti-inflammatory, and antiproliferative properties. This study investigates whether flavonoids, such as quercetin and 2,3-dehydrosilybin, can act as photoactivatable carbon monoxide (CO)-releasing molecules under physiological conditions. CO has been recently recognized as an important signaling molecule. Here, we show that upon direct irradiation, CO was released from both flavonoids in PBS with chemical yields of up to 0.23 equiv, which increased to almost unity by sensitized photooxygenation involving singlet oxygen. Photoreleased CO reduced cellular toxicity caused by high flavonol concentrations, partially restored mitochondrial respiration, reduced superoxide production induced by rotenone and high flavonol levels, and influenced the G0/G1 and G2/M phases of the cell cycle, showing antiproliferative effects. The findings highlight the potential of quercetin and 2,3-dehydrosilybin as CO-photoreleasing molecules with chemopreventive and therapeutic implications in human pathology and suggest their possible roles in plant biology.

## 1 Background

Flavonoids represent a broad and diverse category of natural polyphenolic compounds widely found in plants. Their presence in the human diet has been associated with a range of biological activities, including anti-inflammatory, antioxidant, cardioprotective, hepatoprotective, and anticancer properties.^1,2^ These compounds are believed to be responsible for many of the health benefits associated with a plant-based diet and the enhanced health outcomes observed in vegetarians.^3^ This perception likely contributes to their wide use in the population as nutraceuticals.^4^ Among flavonoids, silymarin flavonolignans, isolated from milk thistle (*Silybum marianum* (L.) Gaertn.), are particularly well-studied for their bioactive properties. Milk thistle fruit extract, silymarin, has been extensively used as a hepatoprotective agent.^5^*In vitro* experimental studies have shown that the individual flavonolignans isolated from silymarin, such as silybins A and B, isosilybin A, silychristin A, silydianin, and 2,3-dehydrosilybins A and B (DHS), exert significant biological effects,^6^ including antioxidant,^7,8^ immunomodulatory,^9^ hepatoprotective,^10^ antiviral,^11,12^ and antiproliferative and anticancer activities.^13−15^

Similarly, quercetin (QCT), a flavonol commonly found in citrus fruits, onion, berries, and green tea, has been investigated in several experimental studies for its potential therapeutic benefits in various types of cancer, and its anticancer effects have been confirmed in some clinical trials.^16,17^

However, the poor bioavailability of flavonoids, their metabolism by the gut microbiota, and extensive phase II conjugation in the human body remain significant challenges limiting their clinical application and biological efficacy.^18^ These factors must be taken into account when using flavonols therapeutically.^12,19^ Despite the problems with the bioavailability of flavonoids, there are increasing efforts to develop new formulations due to their high biological relevance. Quercetin has been reported to be more bioavailable when present in micelles or liposomes, nanosuspensions, or cyclodextrin complexes.^20^ Phosphatidylcholine complexes with silybin such as Siliphos improved bioavailability by enhancing absorption^21^ as well as formulation of quercetin in sunflower lecithin (Quercefit) improved oral absorption resulting in a 20-fold higher plasma concentration of quercetin.^22^ In addition, intravenous administration of silibinin is used as a supportive treatment for liver failure due to amanita mushroom poisoning (injection preparation Legalon SIL, Madaus-Rottapharm, Germany).^19^

Moreover, a comprehensive understanding of the mechanisms underlying the action of flavonols remains elusive, hindering their broader integration into medical practice.

**Scheme 1:**
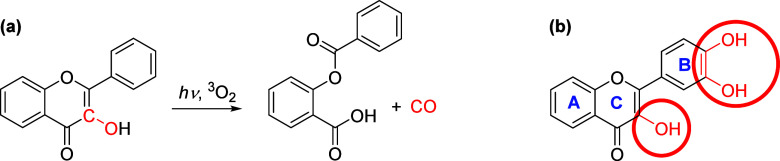
CO Photorelease from 3-Hydroxyflavone (a) and Possible Sites of Photooxygenation (b)

The 3-hydroxyflavone (flavonol) scaffold is the main structural motif of many flavonoids.^1^ Flavonols and their π-extended derivatives can efficiently release carbon monoxide (CO) upon exposure to UV/visible light thanks to oxygenation of the enol 3-OH group (ring C) in the presence of dioxygen (Scheme 1a).^23−30^ Furthermore, we demonstrated that the introduction of a 3′,4′-dihydroxy pattern in the catechol-like ring B of flavonols, which is also a common structural feature of many natural flavonoids, leads to an alternative oxidative photorelease of CO (Scheme 1b).^28^

CO, endogenously produced primarily by the degradation of heme catalyzed by heme oxygenase, serves as a key cellular signaling molecule involved in various physiological processes.^31^ These processes include cytoprotection, apoptosis, regulation of vasoconstriction, oxidative stress, cell cycle control, and inflammatory responses.^32,33^ The promising therapeutic potential of CO has stimulated research and development efforts aimed at designing CO-releasing molecules (CORMs) capable of releasing this gaseous molecule in a controlled manner to mitigate its potential toxicity.^34^

Mitochondria are the primary cell organelles targeted by CO.^35^ Depending on its concentration, CO can either enhance the production of reactive oxygen species (ROS) in the mitochondria or act as an antioxidant and a mild mitochondrial uncoupler.^36,37^ Mitochondria, in particular, produce considerable amounts of superoxide, which contributes significantly to cellular oxidative damage.^38^ Superoxide production is sensitive to the movement of protons across the mitochondrial membrane driven by electrochemical potential; thus, mild uncoupling can significantly attenuate superoxide production.^39^

In addition to the biological effects of CO on human cells and tissues, CO is also a vital molecule in plants, contributing to a plethora of biological processes and protecting against abiotic stress.^40^ It is assumed that CO in plants originates from heme catabolism, and small amounts of CO can arise from lipid peroxidation.^40,41^

Hence, the primary objective of this study was to determine whether common natural flavonoids can function as photoactivatable CORMs (photoCORMs) when exposed to light under physiological conditions. In addition, we aimed to explore the potential role of this CO-releasing process in elucidating some of the observed biological effects associated with flavonoids upon irradiation.

## 2 Materials and Methods

### 2.1 Chemicals

All chemicals were purchased from Merck & Co., Inc. (Rahway, NJ, USA), the organic solvents were purchased from Penta (Prague, Czech Republic), and the cell culture media and reagents were purchased from Biosera (Nuaillé, France) unless otherwise stated. A diastereomeric mixture of silybin AB (ca. 50% silybin A and 48% silybin B) was obtained by treatment of silymarin (Liaoning Senrong Pharmaceuticals, Panjin, China, batch No. 120501) with methanol followed by filtration over a Celite 545 layer. Silybin diastereomers were separated using continuous diastereomeric enzymatic resolution with immobilized lipase B from *Candida antarctica* (Novozyme 435, Novo Nordisk, Copenhagen, Denmark),^42^ yielding silybin A (97%) and silybin B (99%). 2,3-Dehydrosilybin A (95%), 2,3-dehydrosilybin B (98%), 2,3-dehydrosilybin AB (50% 2,3-dehydrosilybin A, 48% 2,3-dehydrosilybin B), and 2,3-dehydrosilychristin A (99%) were prepared by oxidation of silybin A, silybin B, silybin AB, and silychristin A, respectively.^43,44^

### 2.2 Absorption Spectra

UV–vis spectra were measured on an Agilent Cary 8454 UV–vis spectrometer in phosphate-buffered saline (PBS) solutions (5% DMSO, pH = 7.4, *c* = 10 mM, *I* = 100 mM) in 3.0 mL of quartz cells. In order to measure spectra at different pHs, a sodium chloride solution (5% DMSO; *c* = 0.16 M to keep the same ionic strength as that of a PBS solution), in which the pH was adjusted by the addition of HCl or NaOH, was used.

### 2.3 General Procedure for Irradiation in UV Cuvettes

A flavonoid molecule was dissolved in the indicated solvent (3 mL) and placed in a 1 cm quartz PTFE screw-cap cuvette with a stirring rod. The solution was continuously stirred while being exposed to an LED array at a specified wavelength. The progress of the photoreaction was monitored at the specified time intervals by using UV–vis spectroscopy, employing a diode-array spectrophotometer. The reaction conversions were also verified by HPLC (C18-RP column).^28^ When irradiated with rose bengal (RB) as a singlet oxygen sensitizer, the concentration of RB was 5 μM and a 545 nm LED irradiation source was used. The exact masses of flavonols and the products formed upon irradiation were obtained using a TOF triple quadrupole ESI and APCI mass spectrometer working in positive or negative mode coupled with direct inlet or liquid chromatography.

### 2.4 Production of CO in the Dark

Flavonoid samples in the same solutions used for photochemical experiments were left in the dark for a given time, and the CO yields were determined.

### 2.5 Determination of CO Yields

A flavonoid solution in a specified solvent was introduced into sealed gas chromatography (GC) vials equipped with PTFE septa. The vials were irradiated by using an LED array for specified durations. CO was quantified using a GC-headspace instrument equipped with a TIC/MS detector, operating in a SIM mode, and a GC column GC MXT-Msieve 5A PLOT column (30 m, 0.53 mm ID). The instrument was calibrated using the photoreaction of a cyclopropenone photoCORM described elsewhere.^45^

### 2.6 Cell Cultures

The human hepatoblastoma cell line HepG2 (ATCC, Manassas, VA, USA), terminally differentiated hepatic cells derived from a human hepatic progenitor cell line HepaRG (Gibco, Waltham, MA, USA), and the human T-lymphocyte leukemia cell line Jurkat (ATCC, Manassas, VA, USA) were grown in supplemented minimum essential medium (HepG2), William’s medium (HepaRG), or RPMI medium (Jurkat) in a humidified atmosphere containing 5% CO_2_ at 37 °C according to the manufacturer’s instructions. The medium without phenol red (Merck & Co., Rahway, NJ, USA) was used for all irradiation experiments.

### 2.7 CO Quantification

#### 2.7.1 In PBS Buffer

QCT or DHS dissolved in 5% DMSO in PBS buffer (100 μL of 0.4 mM solution) in clear, gastight, septum-sealed vials was irradiated with white light (LED, *I* = 160 mW/cm^2^; TSE, Czech Republic, the wavelength range of 430–500 nm), and the CO production was measured over time by gas chromatography with a reducing gas analyzer (GC/RGA Peak Performer 1, Peak Laboratories, Mountain View, CA, USA) as previously described.^46^

#### 2.7.2 In Cell Medium

HepaRG cells were seeded in a Petri dish (*d* = 6 cm), treated with a solution of QCT or DHS (*c* = 50 μM dissolved in 1% DMSO in colorless medium) and irradiated with white light (LED, *I* = 160 mW/cm^2^) or kept in the dark for 30 min. The control samples contained a medium without QCT or DHS. A medium aliquot (180 μL) was injected into a septum-sealed glass vial purged with a CO-free nitrogen atmosphere containing 20 μL of 30% sulfosalicylic acid at 5 min intervals during the first 30 min and then after 60, 90, and 120 min. CO released into the vial headspace was measured with GC/RGA as previously described^47^ and expressed as nmol of CO per L of medium.

### 2.8 Cytotoxicity Determination

HepaRG cells were seeded in 96-well plates and treated with QCT or DHS in the concentration range of 6.25–400 μM dissolved in colorless MEM or William medium containing 1% DMSO, and irradiated with white light (LED, *I* = 160 mW/cm^2^) for 2 h or kept in the dark for up to 24 h. After incubation, MTT (3-(4,5-dimethylthiazol-2-yl)-2,5-diphenyltetrazolium bromide) reduction assay was performed as previously described.^48,49^

### 2.9 High-Resolution Respirometry

HepaRG or Jurkat cells were seeded in a Petri dish (*d* = 6 cm) and treated with a solution of QCT or DHS (*c* = 50 μM) for 30 min. Samples were irradiated with white light (LED, I = 160 mW/cm^2^) or kept in the dark for the entire incubation period (30 min). After this treatment, HepaRG cells were washed with Hank’s solution, trypsinized, and resuspended in the original treatment solutions, and the final suspension was used for respiration measurement. Jurkat cells were used without previous trypsinization. To determine the degree of cell respiration, the oxygen consumption of living cells was measured using an Oxygraph-2k (Oroboros Instruments GmbH, Austria). Respiration was measured at 37 °C in 2 mL chambers and expressed as the O_2_ flux per 10^6^ cells (pmol s^–1^ 10^–6^ cells); the cell count was measured on Countess automatic cell counter (Invitrogen, USA, Massachusetts). After calibration of the instrument (air calibration, equilibration), we proceeded with the coupling-control protocol for living cells (SUIT-003) using oligomycin (*c* = 2.75 μM), rotenone (*c* = 0.2 μM), and antimycin A (*c* = 5 μM) as inhibitors, and FCCP (titrated by 1 μM steps up to the final concentration of 4–6 μM) as an uncoupler.^50^ The data obtained were analyzed using Datlab software (version 7.4.0.4, Oroboros Instruments).

### 2.10 Mitochondrial Superoxide Production Determination

Superoxide production in living HepaRG and Jurkat cells was determined by using MitoSOX dye (Life Technologies, CA, USA). HepaRG and Jurkat cells were seeded in a Petri dish (*d* = 6 cm) and treated with a solution of QCT or DHS (*c* = 50 μM) for 30 min. Samples were irradiated with white light (LED, *I* = 160 mW/cm^2^) or kept in the dark for the entire incubation time (30 min). After this treatment, cells were washed with Hank’s solution, trypsinized (HepaRG), resuspended in PBS, incubated with MitoSOX dye for 15 min, then centrifuged and resuspended again in PBS. The fluorescence of the stained cells was measured using a flow cytometer (Mindray, Bricyte E6, China).

### 2.11 Fluorescence Microscopy

HepaRG cells were seeded in a 12-well plate and treated with a solution of QCT or DHS (*c* = 50 μM) for 30 min. Samples were irradiated with white light (LED, *I* = 160 mW/cm^2^) or kept in the dark for the entire incubation time (30 min). After incubation, cells were washed with Hank′s solution, incubated with red MitoSOX dye (1 μL/mL) for 15 min, and then visualized using fluorescence microscopy (JuLI Stage Real-Time Cell Imaging System, NanoEntek, South Korea).

### 2.12 Cell Cycle Analysis

HepaRG and HepG2 cells were seeded in a Petri dish (*d* = 6 cm) and treated with a solution of QCT or DHS (*c* = 50 μM) for 30 min. Samples were irradiated with white light (LED, *I* = 160 mW/cm^2^) or kept in the dark during the entire incubation period (30 min). After this treatment, cells were washed with Hank’s solution, trypsinized, and centrifuged, and the pellet was fixed with ice-cold 70% ethanol for at least 2 h. Fixed cells were treated with RNase (130 mg/L) and propidium iodide (Thermo Fisher Scientific, Waltham, MA, USA) solution (100 μmol/L) and analyzed by flow cytometry with MRFlow software (Mindray, BriCyte E6, Shenzen, China).

### 2.13 Statistical Analysis

Normally distributed data are presented as mean ± SD, while non-normally distributed data are presented as median ± interquartile range. Statistically significant differences were determined using the Student *t* test or Mann–Whitney U test, depending on the type of data distribution. An ANOVA test and a Holm–Sidak multiple comparison test were utilized to analyze the differences within the groups. The level of statistical significance was set at *p* ≤ 0.05.

## Results

### CO-Releasing Ability of Different Flavonoids

**Figure 1:**
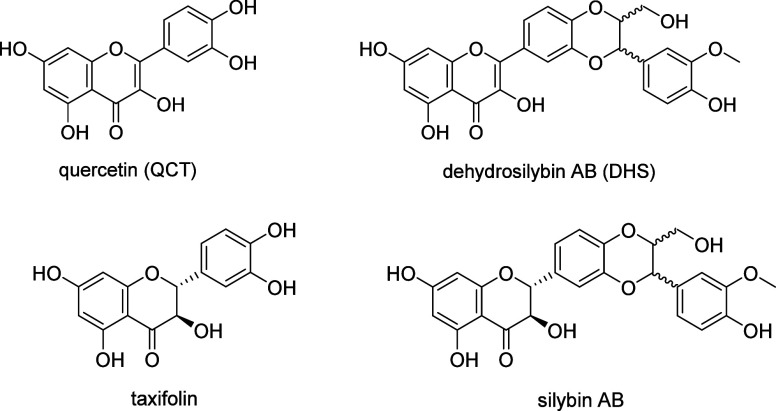
Structures of quercetin (QCT) and 2,3-dehydrosilybin (DHS) and their saturated analogs taxifolin and silybin.

QCT and DHS were selected for an in-depth investigation of their CO-releasing properties (Figure 1) thanks to the favorable CO photorelease properties under physiological conditions.^28^

**Figure 2:**
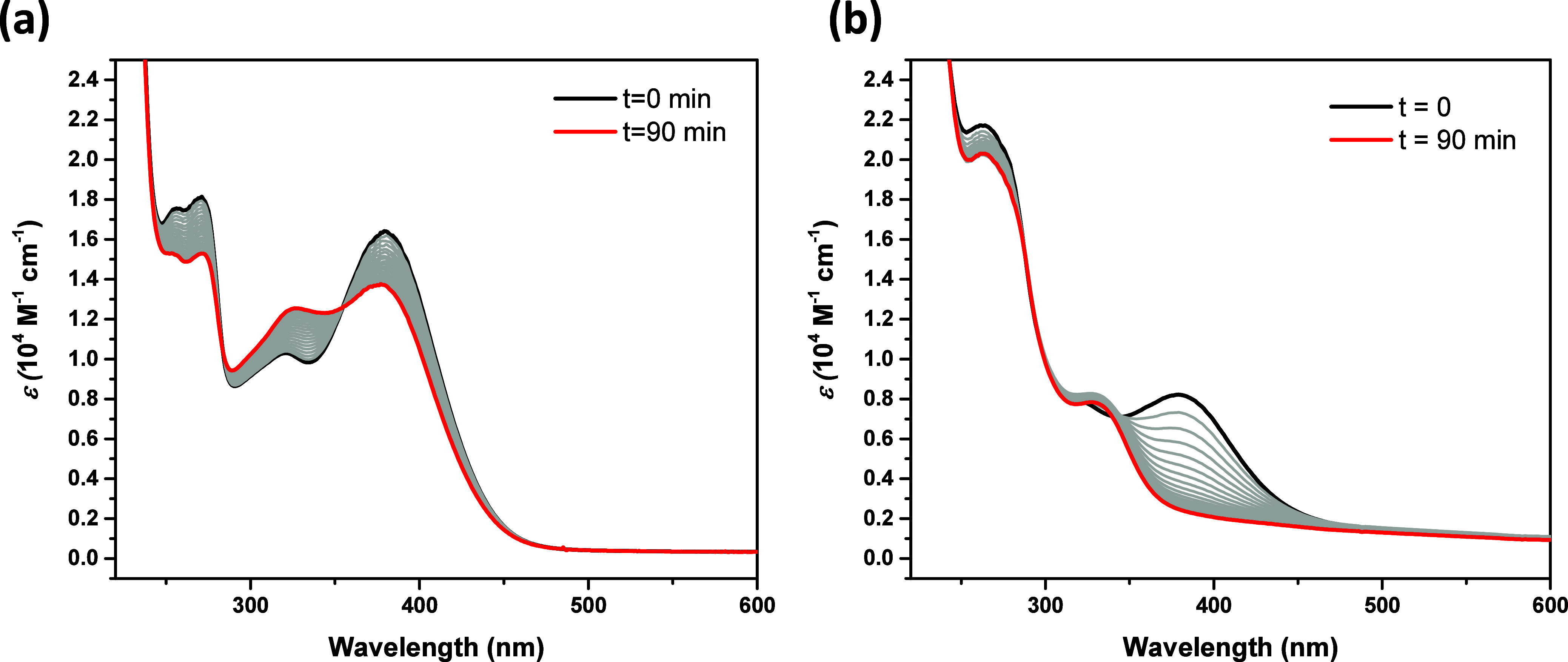
Irradiation of quercetin and 2,3-dehydrosilybin. Irradiation of QCT (a) and DHS (b) in PBS solutions (5% DMSO, pH = 7.4, *c* = 100 mM, *I* = 100 mM). The spectra were recorded every 5 min, and the spectra before (black line) and after (red line) 90 min of irradiation are highlighted.

In this work, most of the spectroscopic and photochemical experiments were conducted in a PBS solution (pH = 7.4, *c* = 10 mM, *I* = 100 mM; 5% DMSO), allowing us to compare the results with those obtained in subsequent cell culture experiments. The absorption maxima (λ_max_) of QCT and DHS in PBS are in the UV region at 378 and 379 nm, respectively (Figure 2). Due to several hydroxyl groups, the acid–base properties of natural flavonols are very complex. The hydroxyls on ring A of QCT are more acidic (p*K*_a_ = 6–7) than those on ring B.^51,52^ For DHS, the 7-OH group has a lower p*K*_a_ (7.4) than the 3-OH group (p*K*_a_ = 8.7).^53^ Therefore, we measured the absorption spectra of QCT and DHS also in sodium chloride aqueous solutions (0.16 M; 5% DMSO; thus keeping the same ionic strength as that in a PBS solution) and titrated them with HCl or NaOH solutions to find out whether the 3-OH group is ionized at pH = 7.4 (Figure S9). It is known that such deprotonation has a significant effect on the main absorption band, which is bathochromically shifted.^54^ After the addition of HCl, only minimal changes in the spectra occurred, while after the addition of NaOH, bathochromic shifts of the main absorption bands occurred, typical of the deprotonation of 3-OH.

Irradiation of both flavonoids at 400 nm (i.e., near their absorption maxima) and also at the tail of the absorption at 450 nm (QCT) resulted in their decomposition (Table 1, Figure 2). It was accompanied by the production of CO in chemical yields that depended on the experimental conditions. Photodegradation at full conversion, and especially after exhaustive direct irradiation (Figures S10 and S11), led to much higher yields (up to 0.2 equiv) than those at lower conversion (50%; 0.06 equiv). After irradiation in a nitrogen atmosphere, no (DHS) or very inefficient (QCT) CO release was observed, as molecular oxygen is essential for photooxidative CO release.^55,56^ CO was detected in small amounts (∼0.01 equiv) in samples kept in the dark for the same period of time. Nonphotochemical CO release was already reported for moderately basic media before^57^ and recently thoroughly investigated by Berreau and co-workers.^58^ The photodegradation quantum yields were found to be higher for DHS (0.042) than for QCT (0.009; Table 1). This is also apparent from the course of irradiation of both flavonoids monitored using UV–vis spectroscopy (Figure 2), where DHS exhibits a degradation more efficient than that of QCT, performed under the same irradiation conditions. The presence of a singlet oxygen quencher (1,4-diazabicyclo[2.2.2]octane, DABCO) increased the CO yield in QCT, as observed before,^28^ while the yield for DHS was suppressed. Direct irradiation of taxifolin and silybin (Figure 1) solutions was not carried out as they show no absorption in the 400–450 nm wavelength range.

**Table 1:** Photochemical Properties of Quercetin (QCT) and 2,3-Dehydrosilybin (DHS)^g^

compd	CO yield (dir)/eq^a^	CO yield (dir, quen)/eq^b^	CO yield (sens)/eq^d^	Φ_d_^f^
QCT	0.06 (50%)	0.23 (100%)	1.0 (100%)	0.009 ± 0.001
	0.10 (100%; 0.11)^28^			
	0.10 (100%)^c^			
	0.18 (exhaustive)^e^			
DHS	0.06 (50%)	0.12 (100%)	0.71 (100%)	0.042 ± 0.001
	0.17 (100%)			
	0.20 (exhaustive)^e^			

a In the absence of the singlet oxygen quencher.

b In the presence of the singlet oxygen quencher (quen, 1,4-diazabicyclo[2.2.2]octane, DABCO;^28^ 10 mM); irradiated at λ_irr_ = 400 nm.

c Irradiated at λ_irr_ = 450 nm (LEDs).

d Upon photosensitization (sens; rose bengal, 5 μM; irradiation at 545 nm) to conversions (%) given in parentheses.

e Exhaustive irradiation 3 times longer than that needed for 100% conversion.

f Quantum yields of flavonol degradation (Φ_d_; λ_irr_ = 385 nm; LEDs); standard deviations of the mean are given.

g In PBS solutions (5% DMSO, pH = 7.4, c = 10 mM, I = 100 mM). Total chemical yields in equivalents (recalculated to the starting material conversion) of released CO upon direct irradiation (dir).

In addition, we investigated the photoreactivity of QCT and DHS toward singlet oxygen produced by sensitization with rose bengal (5 μM, λ_irr_ = 545 nm) used as a singlet oxygen generator. The decomposition of both flavonols was very efficient and led to CO production in very high yields (up to 1 eq; Table 1), which is in accord with our previous findings.^28,56^ We also attempted to identify other major photoproducts of this reaction by HRMS (Figures S1 and S2). For both flavonoids, a salicylic acid derivative, the well-established photoproduct of flavonol photooxygenation,^23,29,56^ along with the products of its dark hydrolysis, was found.

**Figure 3:**
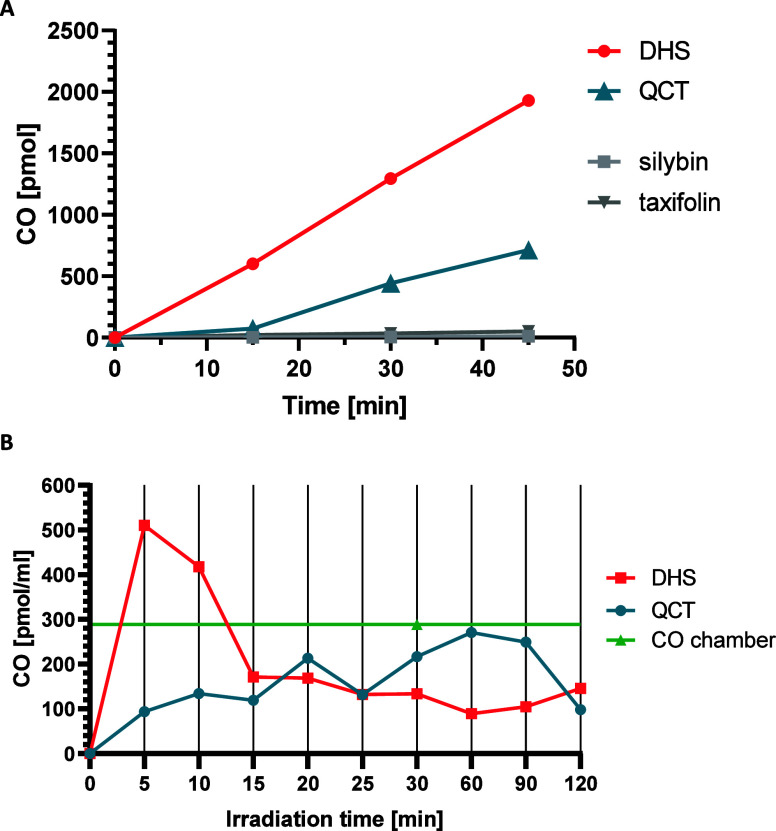
CO-releasing ability of quercetin and 2,3-dehydrosilybin. (A) In PBS buffer, QCT or DHS (100 μL of 0.4 mM solution in PBS buffer with 5% DMSO) was irradiated with white light (LED, *I* = 160 mW/cm^2^) and the liberation of CO into the headspace was determined over time by gas chromatography. Released CO was expressed as pmol of CO released to the vial headspace. (B) In human HepaRG hepatic cells, cells were incubated with QCT or DHS (50 μmol/L in MEM medium with 5% DMSO) and irradiated with white light for 2 h. For the CO chamber experiment, the cells in the medium were exposed to the atmosphere containing 300 ppm CO for 2 h. CO concentration in the medium was measured by gas chromatography and expressed as pmol of CO per mL of medium. CO, carbon monoxide; DHS, 2,3-dehydrosilybin; QCT, quercetin.

In the next set of experiments, we optimized the conditions for *in vitro* studies, where QCT and DHS were irradiated with white light (LED, intensity = 160 mW/cm^2^; wavelength range: 430–500 nm, overlapping with the tails of the absorption spectra of both flavonol derivatives, Figure 2). Meanwhile, irradiation of both QCT and DHS in the presence of atmospheric oxygen leads to the release of CO (Figure 3A); no CO release was observed after irradiation of their saturated analogs taxifolin and silybin, which lack the 2,3-double bond (Figure 2A).^28^ In contrast, no (DHS) or very slow (QCT, Figure S3) CO release was observed upon irradiation in a nitrogen atmosphere, and no release was detected after incubation in darkness.

Subsequently, we investigated whether the addition of QCT or DHS to the HepaRG cell culture could lead to an increase in the CO concentration in the cell medium. Irradiation of cells with white light led to a significant increase in the CO concentration in the medium upon treatment with 50 μM DHS, especially within the first 15 min of irradiation. In contrast, the same concentration of QCT resulted in a less efficient but more sustained increase in the CO concentration (Figure 3B), which is in accord with the measured quantum yield differences using monochromatic light (Table 1). No increase in CO was observed after incubation of cells with QCT or DHS in the dark or after irradiation of cells without flavonoids (Figure S3).

### Effect of CO Released from Irradiated Flavonols on Cell Toxicity

**Figure 4:**
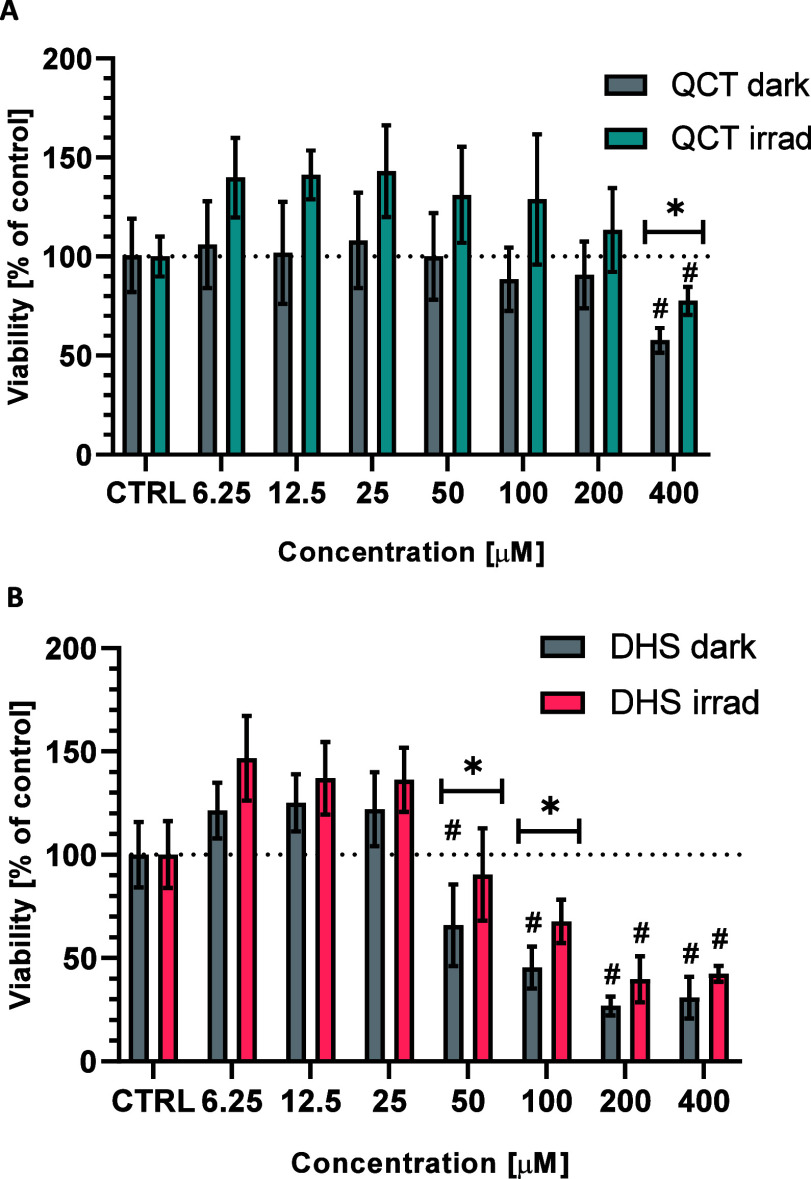
Effect of light irradiation on the viability of human hepatic HepaRG cells exposed to quercetin (A) and 2,3-dehydrosilybin (B). Cells were treated with solutions of QCT or DHS for 24 h in the dark (gray bars) or irradiated for 2 h with white light (LED, *I* = 160 mW/cm^2^) and then incubated for 22 h in the dark (colored bars). *p ≤ 0.05. DHS, 2,3-dehydrosilybin; QCT, quercetin.

To evaluate the potential toxicity of photogenerated CO, HepaRG cells were exposed to QCT or DHS at concentrations ranging from 6.25 to 400 μmol/L for 24 h in the dark or irradiated for 2 h followed by 22 h in the dark. We observed toxicity at high concentrations of flavonols (QCT from 400 μmol/L, DHS from 50 μmol/L). CO released by irradiation attenuated the toxicity induced by QCT and DHS (Figure 4), suggesting a protective effect of CO released by irradiated flavonols.

### Effect of CO Released from Irradiated Flavonols on Mitochondrial Functions

**Figure 5:**
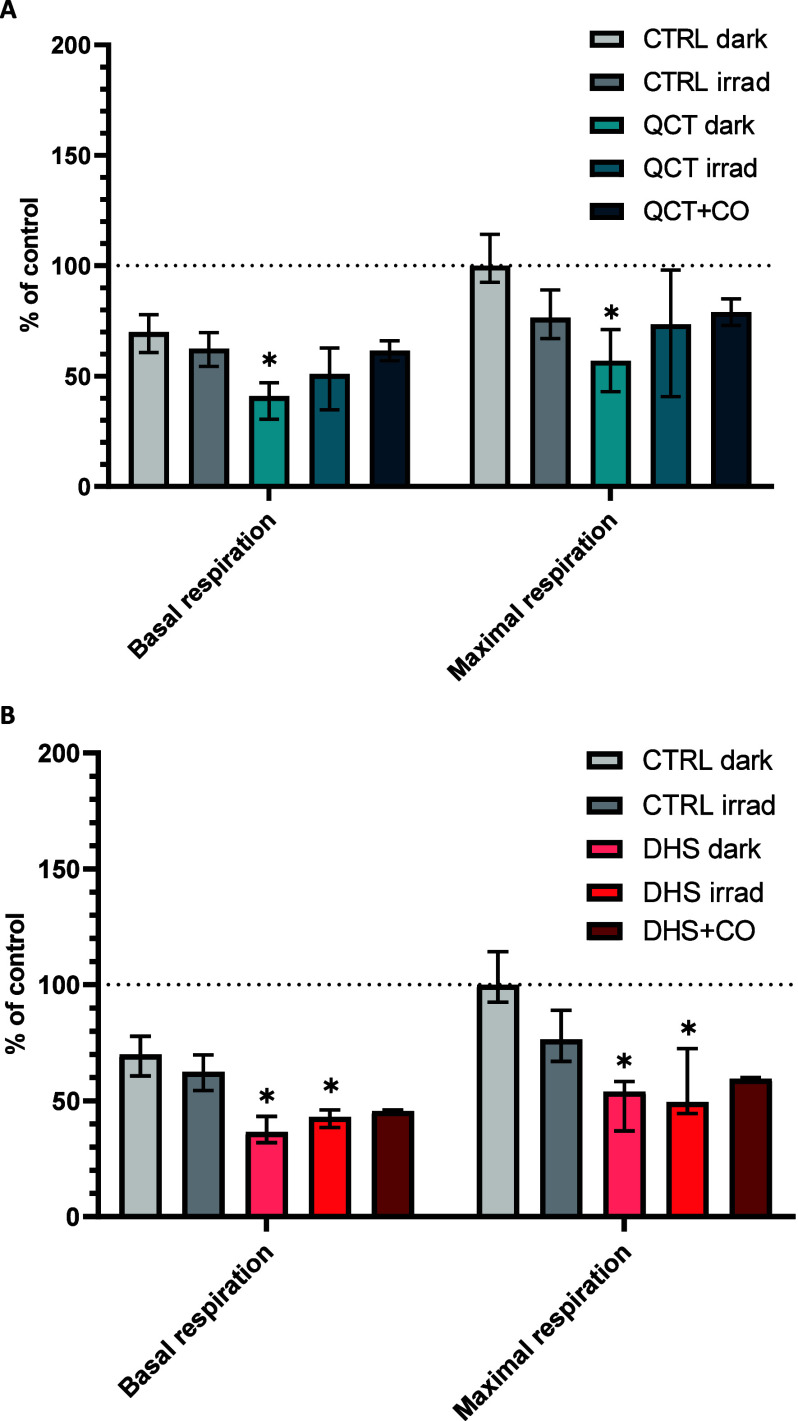
Effect of light irradiation on respiration of human Jurkat cells exposed to quercetin (A) and 2,3-dehydrosilybin (B). Jurkat cells were treated with quercetin and 2,3-dehydrosilybin (50 μM) and irradiated with white light (120 mW·cm^–2^) for 30 min or treated with CO (300 ppm). Basal and maximum respiration (the values expressed as % of the maximum respiration level of untreated controls) were analyzed immediately after incubation. *P-value ≤0.05; *n* ≥ 4.

Given the central role of mitochondria in CO-mediated cell signaling, we examined whether irradiation of cells treated with QCT or DHS affects mitochondrial function in HepaRG and immortalized human T-lymphocyte Jurkat cells. Jurkat cells were selected as a model of suspension cells, allowing us to bypass trypsinization during cell harvest and thus better observe the influence of CO. Using high-resolution respirometry, we examined the basal and maximal respiration rates in Jurkat cells treated with 50 μM QCT or DHS for 30 min. After treatment with 50 μM QCT or DHS, a significant decrease in both basal and maximal respiration was observed. The CO released after irradiation modestly but significantly attenuated this effect in the cells treated with QCT (Figure 5). To confirm that this effect was due to CO, Jurkat cells were incubated with QCT or DHS (50 μM) in a CO chamber containing 300 ppm of CO, a concentration comparable to that measured in the medium after irradiation of flavonols (Figure 3B). CO exposure attenuated the decrease in mitochondrial respiration induced by QCT and DHS in Jurkat cells (Figure 5).

**Figure 6:**
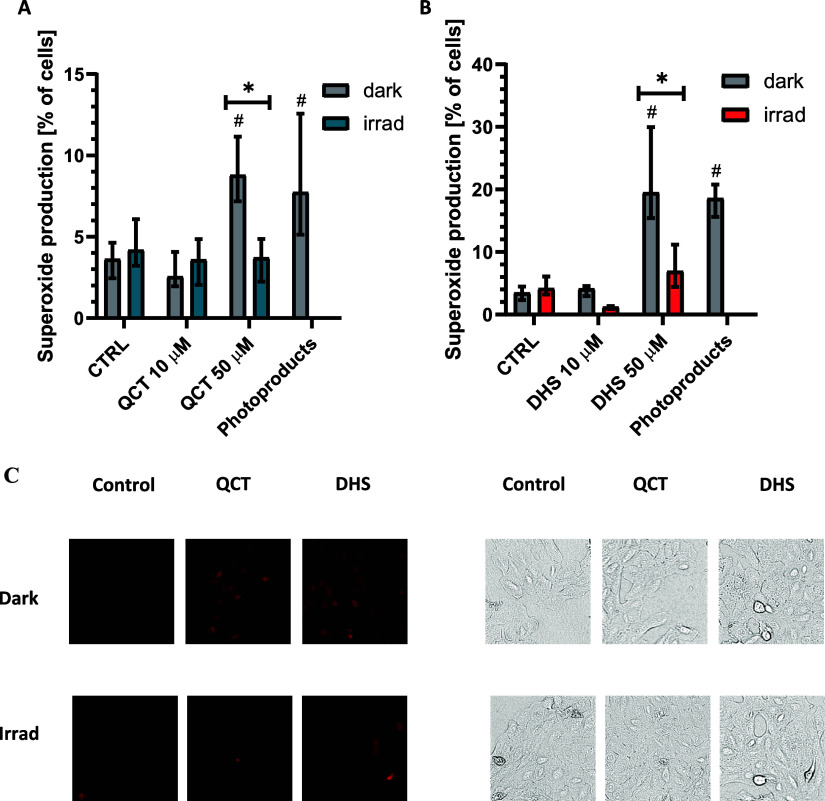
Effect of light irradiation on superoxide production by human hepatic HepaRG cells exposed to quercetin and 2,3-dehydrosilybin. HepaRG cells were exposed to quercetin (QCT) (A) or 2,3-dehydrosilybin (DHS) (B) (50 μmol/L) in the dark or irradiated (irrad) with white light (LED, I = 160 mW/cm^2^) for 30 min. Superoxide production was measured by flow cytometry in live cells using MitoSOX dye. *p ≤ 0.05; *n* ≥ 6. (C) Fluorescence (left panels) and bright-field (right panels) microscopy images of HepaRG cells. After incubation for 15 min with MitoSOX dye, the cells were visualized using white light (bright field) and the RFP channel (red fluorescence) using JuLI Stage Real-Time Cell Imaging System, NanoEntek, South Korea.

Disruption of mitochondrial functions is closely associated with electron leakage from mitochondria, leading to increased superoxide production. We investigated whether treatment with QCT or DHS affects superoxide production and whether the CO generated by irradiation of these flavonols can modulate this effect. Treatment with 50 μM QCT or DHS significantly increased the mitochondrial superoxide concentrations in HepaRG cells. This increase was completely abolished by CO release upon irradiation of the treated cells with white light (Figure 6, Figure S5).

**Figure 7:**
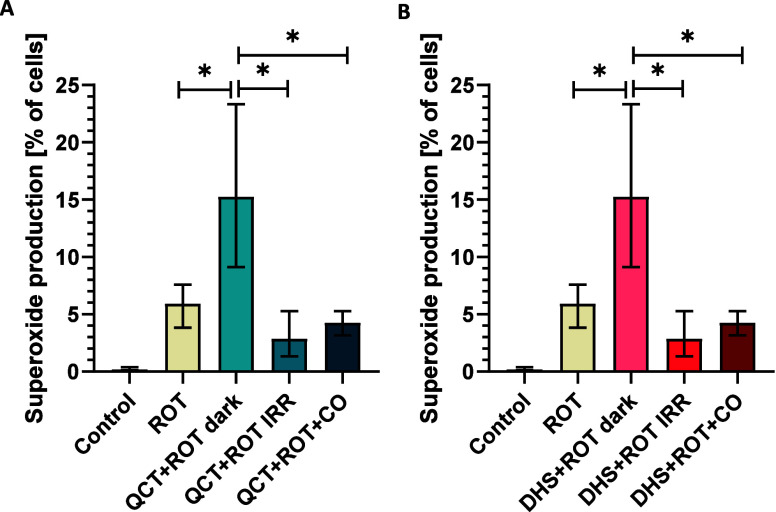
Effect of light irradiation and CO exposure on superoxide production by human Jurkat cells treated with quercetin and 2,3-dehydrosilybin. Jurkat cells were exposed to quercetin (QCT) (A) or 2,3-dehydrosilybin (DHS) (B) (50 μmol/L) and rotenone (ROT, 10 μM) in the dark or irradiated with white light (LED, *I* = 160 mW/cm^2^) or treated with CO (300 ppm) for 30 min. Superoxide production was measured by flow cytometry in live cells using MitoSOX dye. *p ≤ 0.05; *n* ≥ 6.

To ensure that this effect was not due to a decrease in the flavonol concentration during irradiation, we incubated the cells with lower concentrations of QCT and DHS as well as with a 50 μM solution of a mixture of photoproducts obtained after 30 min of irradiation of QCT and DHS in colorless MEM medium. Although incubation of the cells with 10 μM QCT and DHS solutions resulted in a significant decrease in superoxide production, treatment with photoproducts from irradiated solutions of QCT or DHS caused an increase in superoxide production comparable to that observed with QCT or DHS alone (Figure 7).

To further investigate the role of flavonol irradiation on superoxide production, Jurkat cells were treated with QCT or DHS in combination with rotenone, a complex I inhibitor that induces electron leakage and superoxide production. The combined treatment with QCT and DHS resulted in an increase in the level of superoxide production. However, irradiation with white light or incubation in a CO atmosphere completely abolished this effect, further confirming the modulatory role of CO in superoxide production (Figure 7, Figure S6).

### Effect of CO Released from Irradiated Flavonols on Cell Proliferation

**Figure 8:**
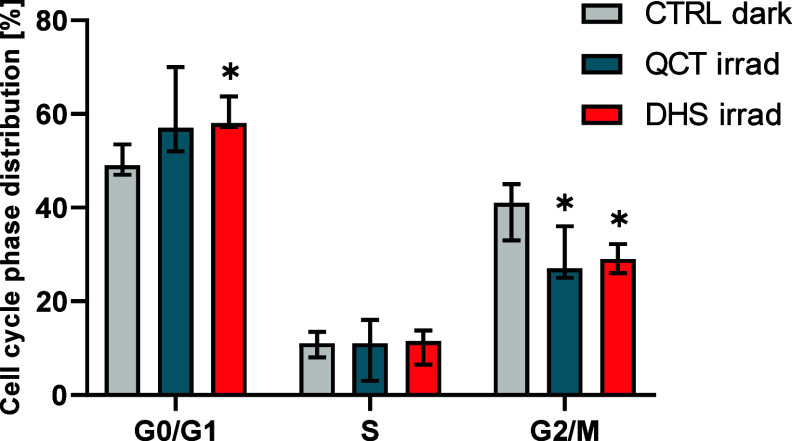
Effect of light irradiation on the cell cycle of human hepatic HepG2 cells exposed to quercetin and 2,3-dehydrosilybin. HepG2 cells were exposed to quercetin (QCT) or 2,3-dehydrosilybin (DHS) (50 μmol/L) and irradiated with white light (LED, I = 160 mW/cm^2^) for 2 h. The cell cycle was measured by flow cytometry after another 22 h in the dark. *p ≤ 0.05; *n* ≥ 6.

We also investigated the influence of CO released during irradiation on the cell proliferation. No significant changes in proliferation were observed after treatment of HepaRG cells with QCT or DHS without or with irradiation (Figure S7). As this cell line is considered to be slowly dividing and relatively differentiated, we carried out similar experiments with a less differentiated HepG2 hepatoblastoma cell line. Our results showed that irradiation of HepG2 cells treated with QCT or DHS significantly inhibited cell division, as evidenced by an increase in G0/G1 phases, particularly in the case of DHS. Additionally, there was a concomitant decrease in the G2/M cell cycle phase after treatment with both QCT and DHS compared with untreated controls (Figure 8). Notably, no significant alterations were observed when HepG2 cells were treated with QCT or DHS in the dark or when the control medium was irradiated without the presence of an active substance (Figure S8).

## 4 Discussion

Despite the well-documented benefits of flavonols, including QCT and DHS, for human health, the exact mechanisms underlying these pleiotropic effects remain incompletely understood. A common structural feature of flavonols is the 3-hydroxyflavone backbone, which has the potential to release CO upon exposure to ultraviolet (UV) light (Scheme 1).^25,26^ Blocking the 3-OH group turned off this photoreaction. Nevertheless, 3-methoxyflavone undergoes intramolecular cyclization as the main photodegradation pathway.^59^

The photochemistry of simple flavonols involves several reaction pathways that are influenced by pH as they can exist in both acid (3-hydroxy group; p*K*_a_ ∼ 8.7)^51^ and base (enolate) forms in polar protic solvents. The acid form of the parent flavonol reacts via an excited triplet state formed by excited-state intramolecular proton transfer (ESIPT)^60^ and subsequent intersystem crossing, with ground-state O_2_ via an endoperoxide intermediate that rearranges to give CO and salicylic acid ester.^23,56^ The conjugate base of flavonol oxidatively photoreleases CO by an analogous mechanism or the reaction of the ground-state flavonol with singlet oxygen produced by the flavonol triplet-state sensitization.^23,28^

Based on previous^28,51,52^ and current findings (Figure S9), we conclude that one of the most acidic OH groups on the A ring must be deprotonated in PBS (5% DMSO, pH = 7.4). However, the equilibrium concentration of the conjugate base related to the 3-OH group is not significant as the absorption spectra of PBS and the acidified saline solution are essentially the same. The absorption maxima of QCT and DHS (378 and 379 nm, respectively; Figure 2) are very close due to the common 5,7-dihydroxyflavonol basic chromophore, while the substitution on the (phenyl) ring B affects the absorption profile only at shorter wavelengths.

Direct irradiation of both compounds at 400 nm resulted in the formation of side-photoproducts and the simultaneous release of CO in chemical yields up to 0.2 equiv, which was found to be dependent on the reaction conversions (Table 1). In the presence of a singlet oxygen trap (DABCO), the yields increased for QCT (0.23 equiv) but decreased for DHS (0.12). As we demonstrated recently, in addition to photooxygenation of the enol 3-OH group, CO is generated via photooxidation of the catechol group of ring B.^28^ From this perspective, the QCT and DHS structures represent fundamentally different substrates, one of which (QCT) has both photooxidation centers (Scheme 1b), while DHS has only one (the catechol hydroxy groups are blocked as two ether bonds). The catechol group was shown to be the main source of CO upon direct irradiation of QCT in PBS, while the 3-OH group becomes the CO source in the presence of a quencher.^28^ Trapping singlet oxygen not only alters the oxygenation mechanism but also suppresses the unproductive degradation pathways. Photosensitization only enhances the catechol oxidation mechanism, which is the dominant photooxygenation pathway during exhaustive direct irradiation (Table 1).

Although both QCT and DHS photorelease CO with almost the same chemical yields, the photochemical degradation efficiency is significantly higher for DHS. It was shown that after ESIPT, the rotation of the bond between the B and C rings is responsible for the ultrafast nonradiative QCT decay and, thus, a very low fluorescence quantum yield.^61^ We hypothesize that the large B-ring substituent in DHS may suppress this channel and thereby increase the photoreaction quantum yield, but further experiments would be needed to confirm this assumption.

Our results demonstrate that CO production from flavonols must depend on the cellular microenvironment and oxidative status. This is of great importance as submicromolar to low micromolar concentrations of CO have been shown to protect cells from reactive oxygen species.^62^ Averilla et al.^63^ demonstrated that CO derived from the heme oxygenase reaction or administered via CORM-2 treatment protects human keratinocytes from UVB-induced oxidative stress. Therefore, photoreleased CO could contribute to the protection of cells against UV irradiation, an effect previously described for flavonoids.^64^

Notably, the irradiation of QCT or DHS in the cell medium also leads to a measurable increase in the CO concentrations. It is apparent from the absorption spectra of QCT and DHS that these compounds absorb light within the blue part of the spectrum, so that a photoreaction can take place at wavelengths up to about 470 nm. Given the seasonal and daily variations in blue light exposure and the resulting CO photoproduction from flavonol-containing cells, it is plausible to speculate that CO may function as a biological clock, coordinating cellular processes with environmental light cycles. However, further experiments are required to support this hypothesis.

Flavonols exhibit a concentration-dependent effect on the oxidative stress. *In vitro* studies have shown that they can exert potent antioxidant properties at low to moderate concentrations by scavenging reactive oxygen species (ROS) and reducing superoxide levels. This antioxidant effect is primarily attributed to the ability of flavonols to donate electrons and neutralize free radicals, thereby protecting cells from oxidative damage.^65^ However, in high concentrations, flavonols can surprisingly induce oxidative stress by promoting the generation of superoxide and other ROS.^66^ Consistent with these findings, we observed cellular toxicity for QCT and DHS at concentrations above 400 and 50 μM, respectively. However, these concentrations are not biologically relevant *in vivo*. Notably, the CO released by irradiation significantly attenuated this toxicity, suggesting a cellular protective effect of the released CO. These results are consistent with the published data indicating the beneficial effects of low CO concentrations.^33^

In biological systems, CO binds preferentially to transition metals, with iron being the most significant.^67^ Beyond hemoglobin, iron is also an essential component of enzymes such as soluble guanylate cyclase (sGC), nitric oxide synthase (NOS), and mitochondrial heme protein cytochrome c oxidase (COX), which serve as intracellular binding targets for CO.^62^ Depending on the concentration of CO, COX activity can either be induced or suppressed, subsequently influencing cellular respiration and superoxide production.^68^ In our study using HepaRG and Jurkat cells, we found that 50 μM QCT and DHS significantly reduced cellular respiration. When mitochondrial respiration is inhibited or slowed, the accumulation of reduced intermediates (e.g., NADH, FADH_2_) can increase the potential for electron leakage, especially from complexes I and III, leading to superoxide production.^69^ Accordingly, we observed an increase in superoxide production after the treatment of cells with rotenone and 50 μM QCT or DHS, while lower concentrations (10 μM) of flavonols did not induce this effect. Importantly, the CO released from flavonol irradiation mitigated the decrease in mitochondrial respiration and the increase in superoxide production. More consistent data were obtained with suspension Jurkat cells compared to adherent HepaRG cells as Jurkat cells did not require trypsinization and could be incubated with flavonol and CO-containing medium throughout the experiment. These results suggest that released CO plays an important role in maintaining mitochondrial function and modulating cellular responses to ROS *in vitro.* To further validate whether these results are genuinely due to the presence of CO, we incubated cells treated with QCT and DHS in a CO chamber at 300 ppm of CO, a concentration comparable to that obtained by flavonol irradiation. Remarkably, we observed a similar improvement in mitochondrial respiration or superoxide production to that with flavonol irradiation.

It has also been suggested that CO influences cell proliferation.^70^ Therefore, we investigated the effect of QCT and DHS irradiation on the cell cycle. No significant effect was observed in HepaRG cells, which are relatively differentiated and divide slowly. We also tested the more dedifferentiated HepG2 cell line. Irradiation of HepG2 cells treated with QCT or DHS significantly increased the G0/G1 phase and simultaneously decreased the G2/M cell cycle phase, suggesting that a cell-specific antiproliferative effect of CO is consistent with the previously reported anticancer effects.^71^

In summary, this study has shown that QCT and DHS release CO within cells upon irradiation, which significantly influences various cellular processes. In particular, the release of CO considerably affects mitochondrial functions, superoxide production, and cell cycle regulation. These effects are crucial for explaining the beneficial properties of flavonols observed *in vitro*, including their potential protection against UV irradiation as well as their antioxidant and antiproliferative effects. Our findings suggest that these flavonoids could be used as CO-releasing molecules; however, further in-depth studies are needed to evaluate these effects *in vivo*. Since flavonoids are present in high concentrations in plants and are exposed to sunlight, our results may stimulate research on the role of CO in plant signaling and potentially provide new insights into plant biology.

## Supporting Information Available

Figure S1: HR-MS (ESI^+^) of quercetin; Figure S2: HR-MS (ESI^–^) of 2,3-dehydrosilybin; Figure S3: CO-releasing ability of quercetin and 2,3-dehydrosilybin; Figure S4: release of flavonoid-derived CO to cell medium; Figure S5: superoxide production from HepaRG cells: the effect of QCT and DHS irradiation; MitoSox staining; Figure S6: superoxide production from Jurkat cells: the effect of QCT and DHS irradiation; Figure S7: the effect of light irradiation on the cell cycle of human hepatic HepaRG cells exposed to quercetin and 2,3-dehydrosilybin; Figure S8: the effect of light irradiation on the cell cycle of human hepatic HepG2 cells exposed to quercetin and 2,3-dehydrosilybin; Figure S9: titration of quercetin and 2,3-dehydrosilybin in sodium chloride solutions; Figure S10: photodegradation kinetics of quercetin; Figure S11: photodegradation kinetics of 2,3-dehydrosilybin (PDF)

## List of abbreviations

CO: carbon monoxide
CORMs: CO-releasing molecules
COX: mitochondrial cytochrome c oxidase
DHS: 2,3-dehydrosilybins A and B
GC: gas chromatography
GC/RGA: gas chromatography with a reducing gas analyzer
MTT: (3-(4,5-dimethylthiazol-2-yl)-2,5-diphenyltetrazolium bromide)
NOS: nitric oxide synthase
photoCORMS: photoactivatable CO-releasing molecules
QCT: quercetin
RB: rose bengal
ROS: reactive oxygen species
sGC: soluble guanylate cyclase
UV: ultraviolet
