# Supplementary material for: Carbon Monoxide-Releasing Activity of Plant Flavonoids
Source: J Agric Food Chem. 2024 Dec 31;73(2):1308–18. doi: 10.1021/acs.jafc.4c09069 (PMC11741109; doi:10.1021/acs.jafc.4c09069)
Supplement: Supplementary file 1 — jf4c09069_si_001.pdf [file jf4c09069_si_001.pdf]

SUPPORTING INFORMATION

Carbon monoxide-releasing activity of plant flavonoids

Lucie Muchová[1*], Mária Šranková[1], Sriram Balasubramani[1], Panshul Mehta[1], Dafni Vlachopoulou[1], Akshat Kapoor[1], Andrea Ramundo[2,3], Yann Anton Jézéquel [b,c], Igor Božek[2,3], Martina Hurtová[4], Petr Klán[2,3], Vladimír Křen[4], Libor Vítek[1]

[1] Institute of Medical Biochemistry and Laboratory Diagnostics, and 4th Department of Internal Medicine, General University Hospital in Prague and 1st Faculty of Medicine, Charles University, Na Bojišti 3, 12108 Prague 2, Czech Republic

[2] Department of Chemistry, Faculty of Science, Masaryk University, Kamenice 5, 62500, Brno, Czech Republic.

[3] RECETOX, Faculty of Science, Masaryk University, Kamenice 5, 62500, Brno, Czech Republic.

[4] Institute of Microbiology of the Czech Academy of Sciences, Laboratory of Biotransformation, Vídeňská 1083, Prague 4, CZ 14200, Czech Republic.

*Corresponding author: lucie.muchova@lf1.cuni.cz

Table of contents

Figure S1. HR-MS (ESI^+) of quercetin

Figure S2. HR-MS (ESI^-) of 2,3-dehydrosilybin

Figure S3. CO-Releasing ability of quercetin and 2,3-dehydrosilybin

Figure S4. Release of flavonoid-derived CO to cell medium

Figure S5. Superoxide production from HepaRG cells: the effect of QCT and DHS irradiation. MitoSox staining

Figure S6. Superoxide production from Jurkat cells: the effect of QCT and DHS irradiation

Figure S7. The effect of light irradiation on the cell cycle of human hepatic HepaRG cells exposed to quercetin and 2,3-dehydrosilybin

Figure S8. The effect of light irradiation on the cell cycle of human hepatic HepG2 cells exposed to quercetin and 2,3-dehydrosilybin

Figure S9. Titration of quercetin and 2,3-dehydrosilybin in sodium chloride solutions

Figure S10. Photodegradation kinetics of quercetin

Figure S11. Photodegradation kinetics of 2,3-dehydrosilybin

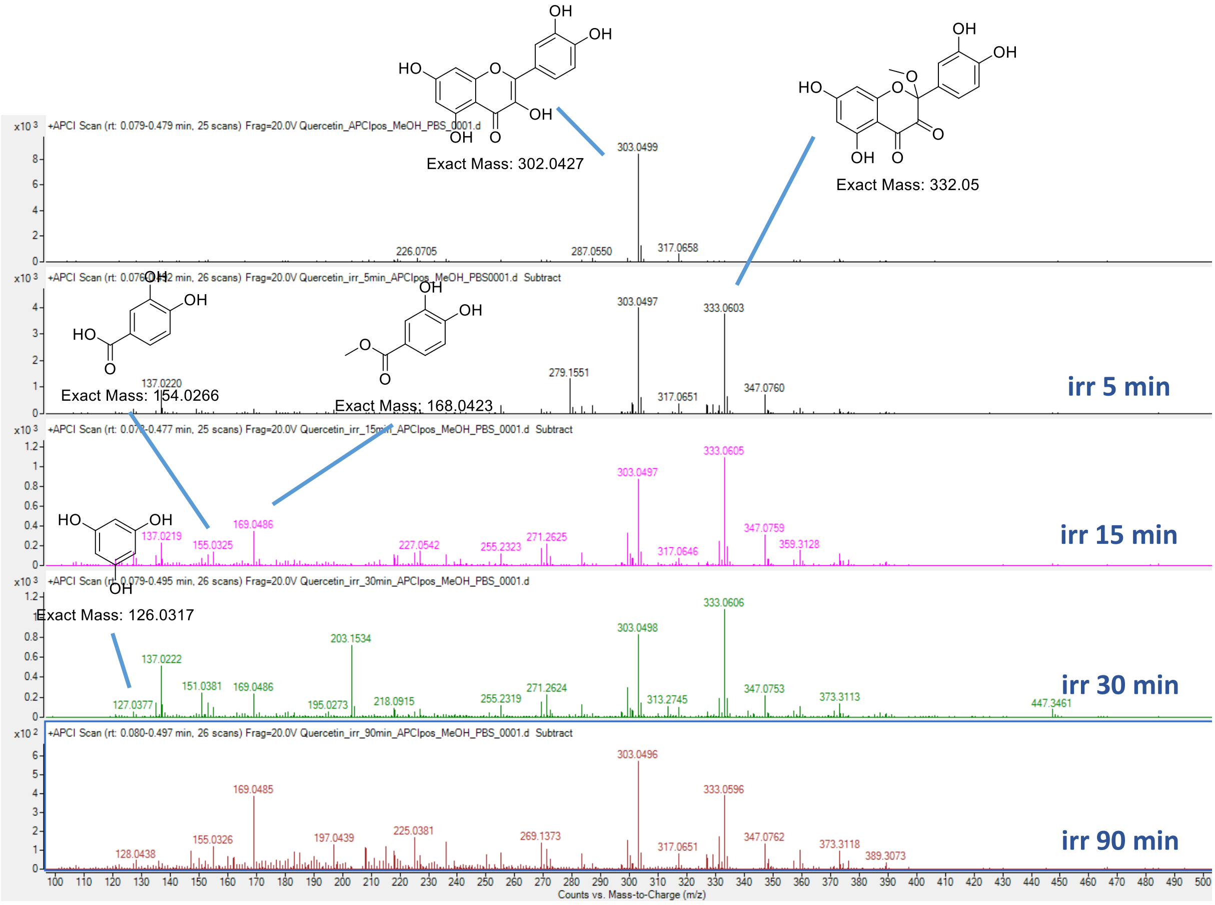

Figure S1. HR-MS (ESI⁺) of quercetin. HR-MS (ESI⁺) of a solution of quercetin (300 µM) and rose bengal (5 µM) in methanol-PBS (3:7 v/v) upon irradiation at 545 nm for different amounts of time.

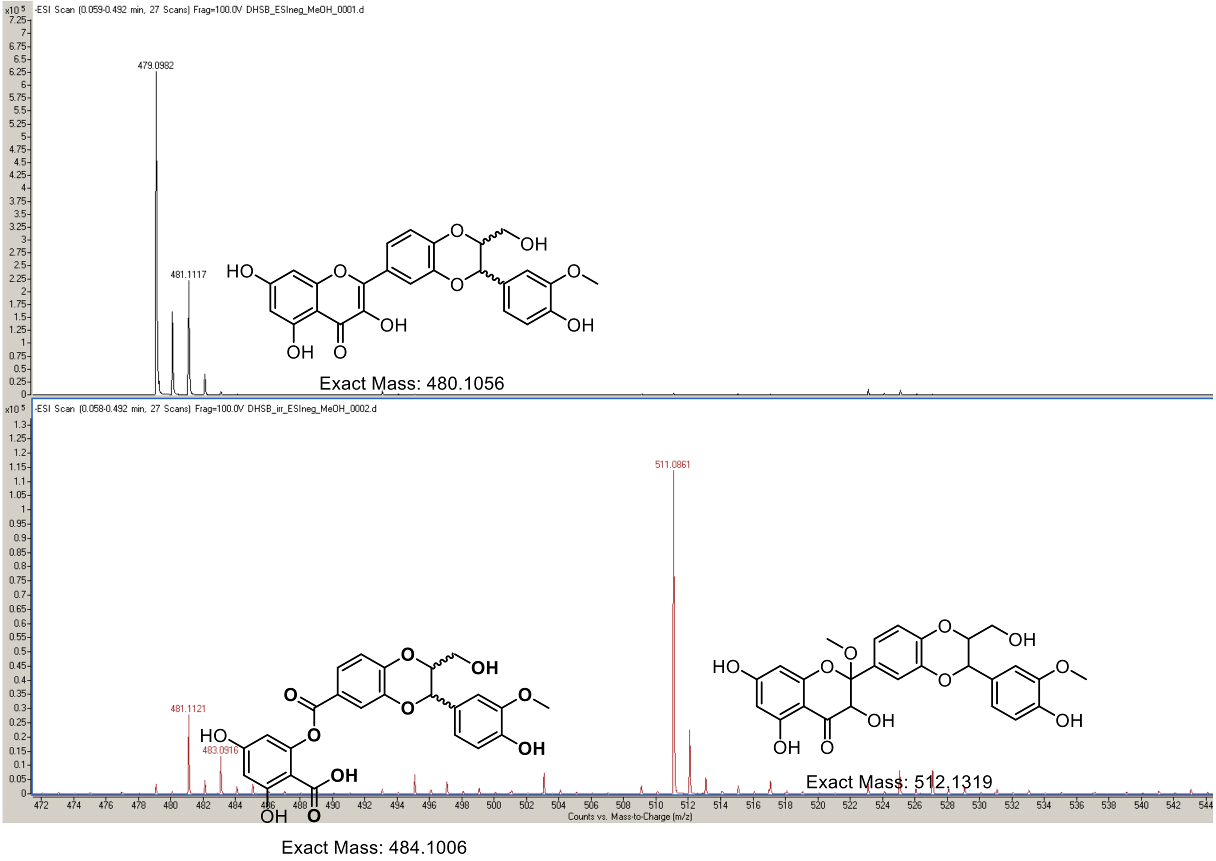

Figure S2. HR-MS (ESI⁻) of 2,3-dehydrosilybin. HR-MS (ESI⁻) of a solution of 2,3-dehydrosilybin (200 µM) and rose bengal (5 µM) in methanol-PBS (1:4 v/v) upon irradiation at 545 nm.

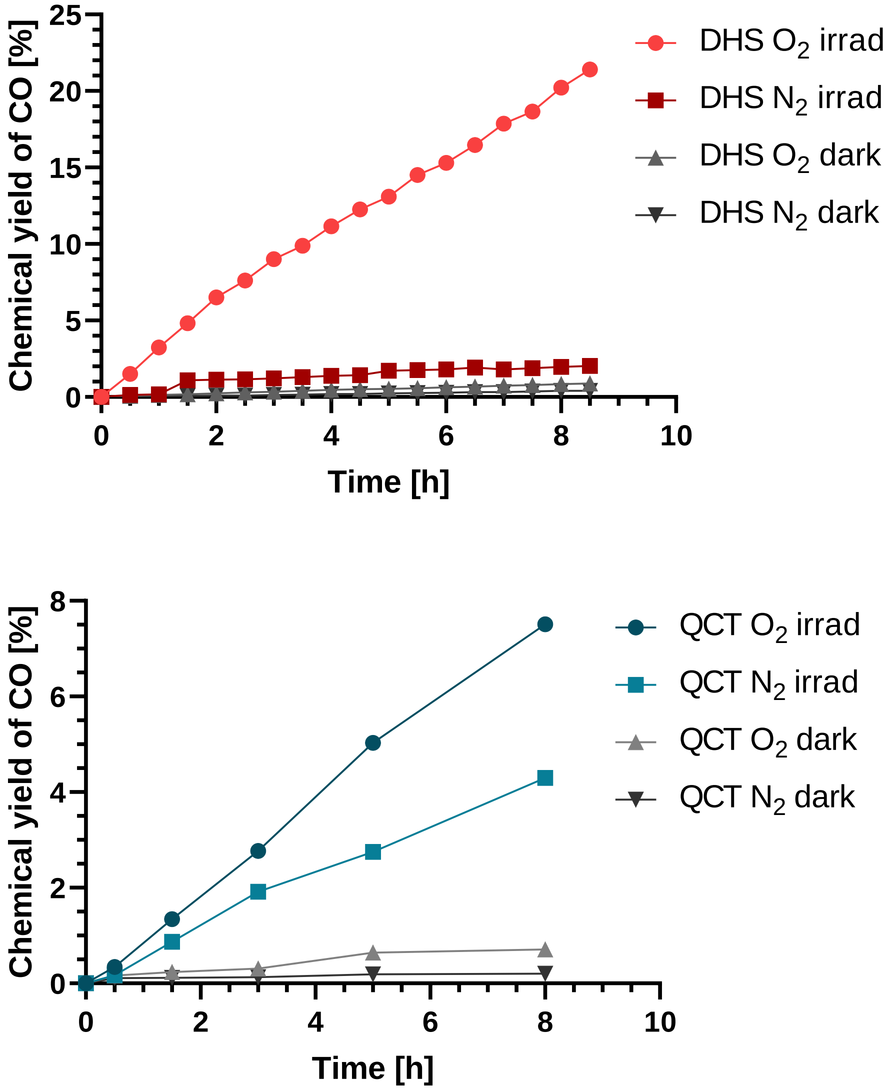

Figure S3. CO-Releasing ability of quercetin and 2,3-dehydrosilybin. QCT or DHS (100 µL of 0.4 mM solution in PBS buffer with 5% DMSO) was irradiated with white light (LED, I = 160 mW/cm^2) with synthetic air (O_2) or N_2 atmosphere (N_2) and CO liberated into the headspace was measured over time by gas chromatography. Released CO was expressed as % of the original compound concentration.

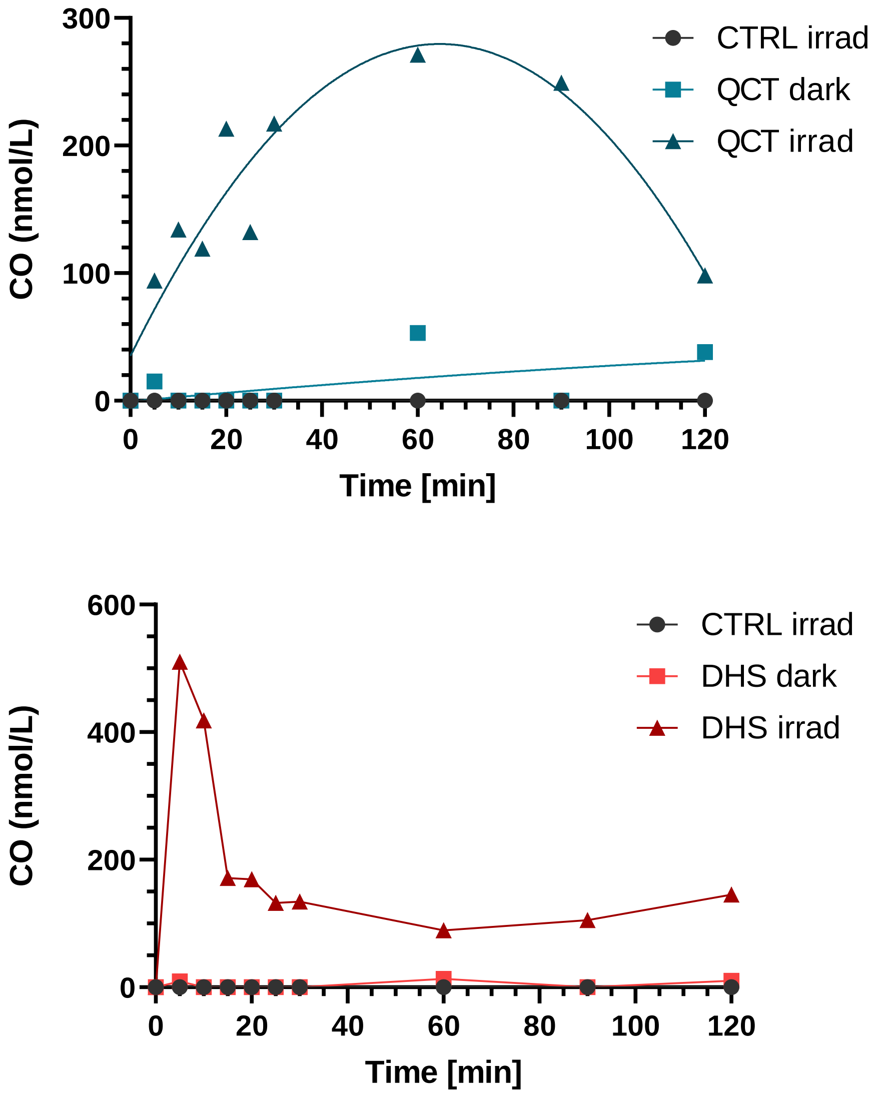

Figure S4. Release of flavonoid-derived CO to cell medium. Hepatic HepaRG cells were incubated with QCT or DHS (50 µmol/L in MEM medium with 5% DMSO) and irradiated with white light (LED, I = 160 mW/cm^2) or kept in the dark for 2 h. Control cells were treated with MEM medium with only 5% DMSO. CO concentration in the medium was measured by gas chromatography and expressed as nmol CO per L of medium.

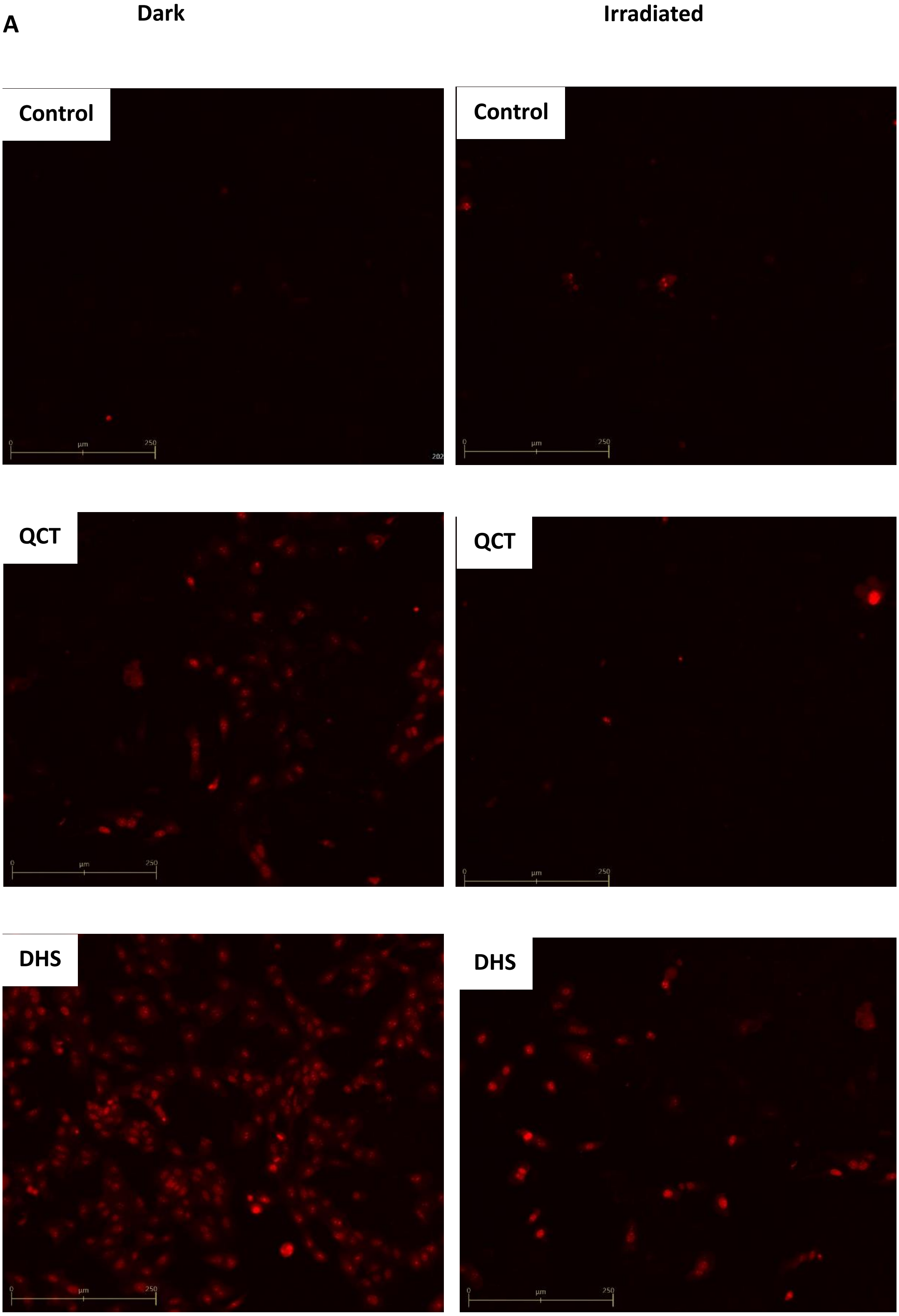
A
Dark
Irradiated
Control
Control
QCT
QCT
DHS
DHS

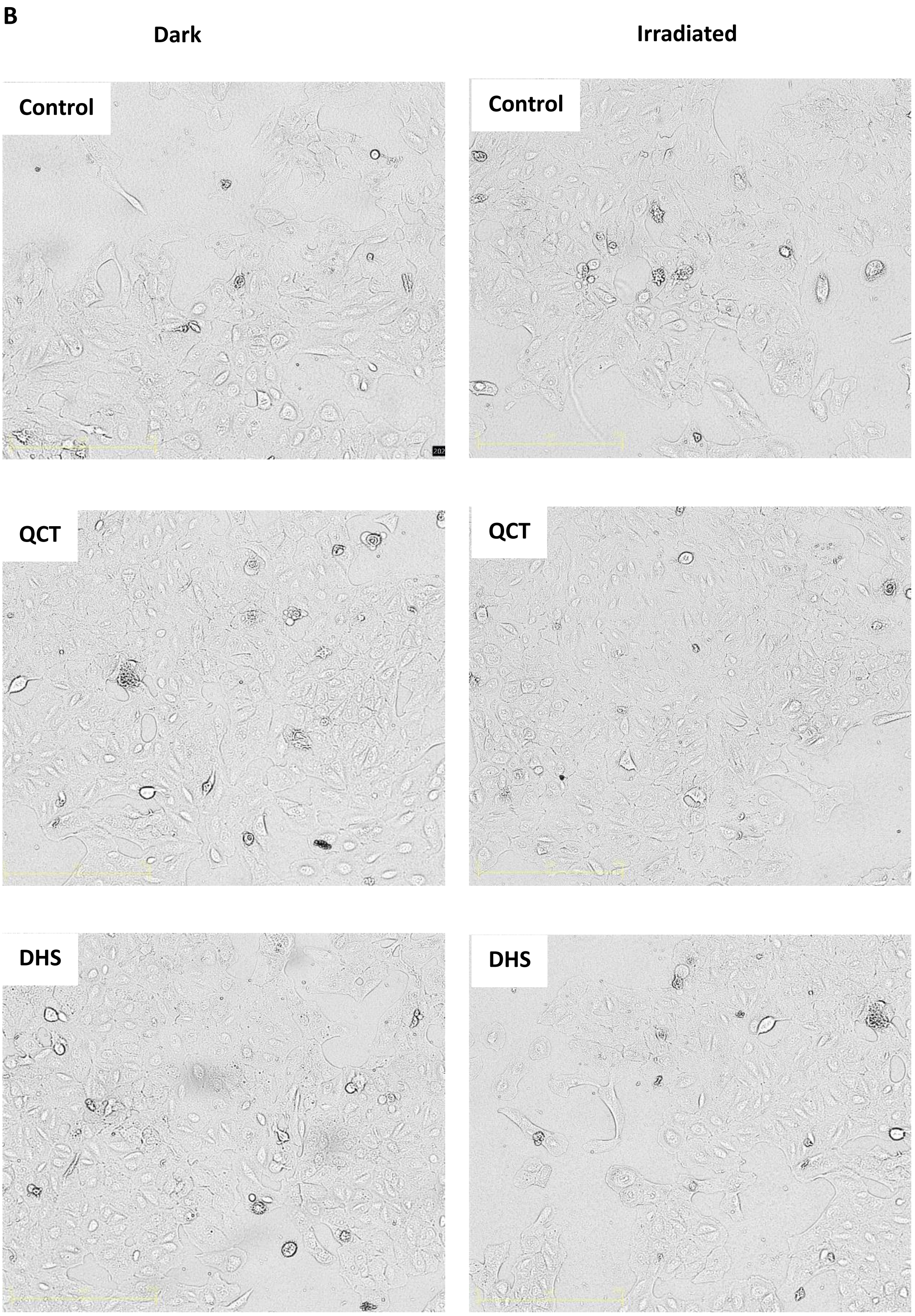

Figure S5. Superoxide production from HepaRG cells: the effect of quercetin and 2,3-dehydrosilybin irradiation. MitoSox staining. Fluorescence (**A**) and bright-field (**B**) microscopy images of HepaRG cells treated with QCT and DHS (50 µM) for 30 min with or without irradiation with white light (LED, I = 160 mW/cm^2). After incubation for 15 min with

MitoSOX dye, the cells were visualized using white light (bright field) and an RFP channel (red fluorescence) using JuLI Stage Real-Time Cell Imaging System, NanoEntek, South Korea. Control cells were incubated in a Williams medium without QCT or DHS.

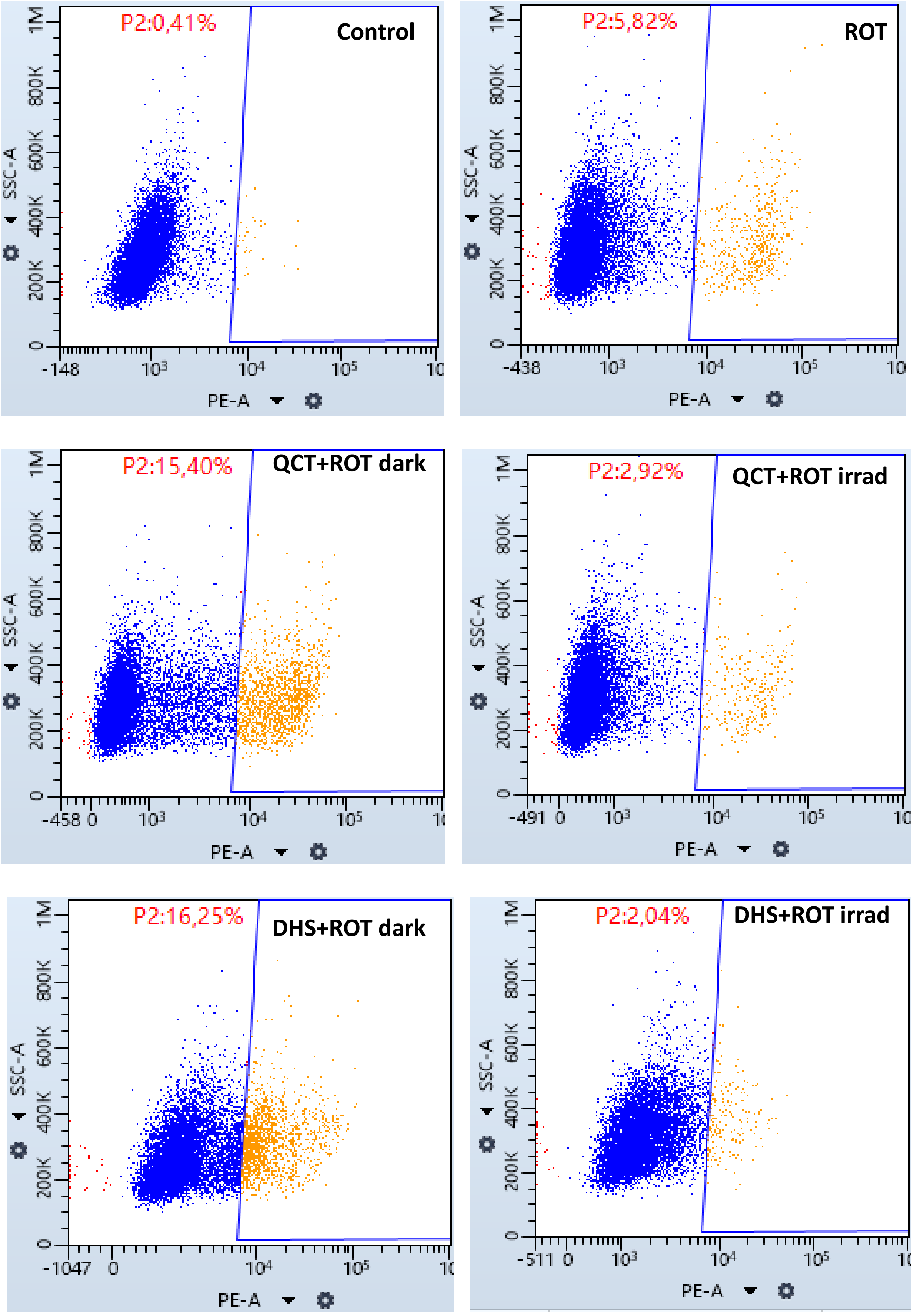

Figure S6. Superoxide production from Jurkat cells: the effect of quercetin and 2,3-dehydrosilybin irradiation. Jurkat cells were exposed to quercetin (A) or 2,3-dehydrosilybin (B) (50 µmol/L) and rotenone (ROT, 10 µM) in dark or irradiated with white light (LED, I = 160 mW/cm^2) for 30 min. Superoxide production was measured by flow cytometry in live cells using red MitoSOX dye. * $p \leq 0.05$; n ≥ 6. Representative figures from flow cytometry.

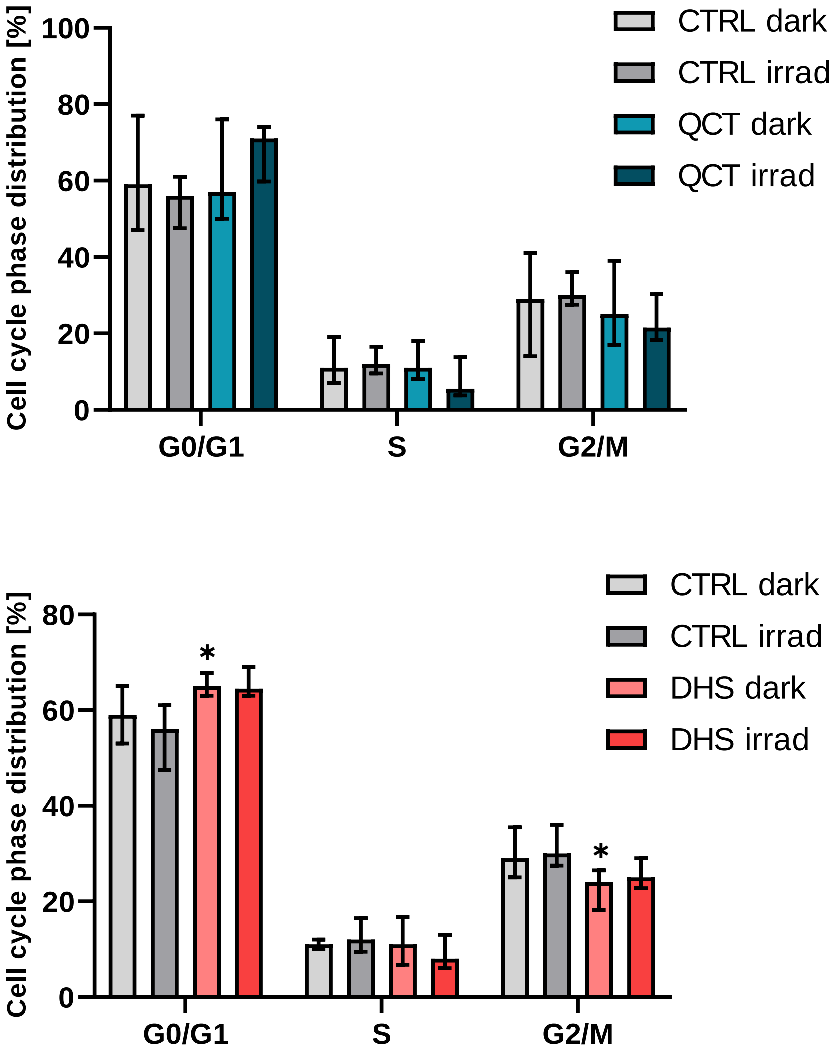

Figure S7. The effect of light irradiation on the cell cycle of human hepatic HepaRG cells exposed to quercetin and 2,3-dehydrosilybin. HepaRG cells were treated with quercetin or 2,3-dehydrosilybin (50 µmol/L) and kept in the dark or irradiated with white light (LED, I = 160 mW/cm^2) for 2 h. Control cells were treated with vehicle (1% DMSO) in the medium. The cell cycle was measured by flow cytometry after another 22 h in the dark. * $p \leq 0.05$; n ≥ 6

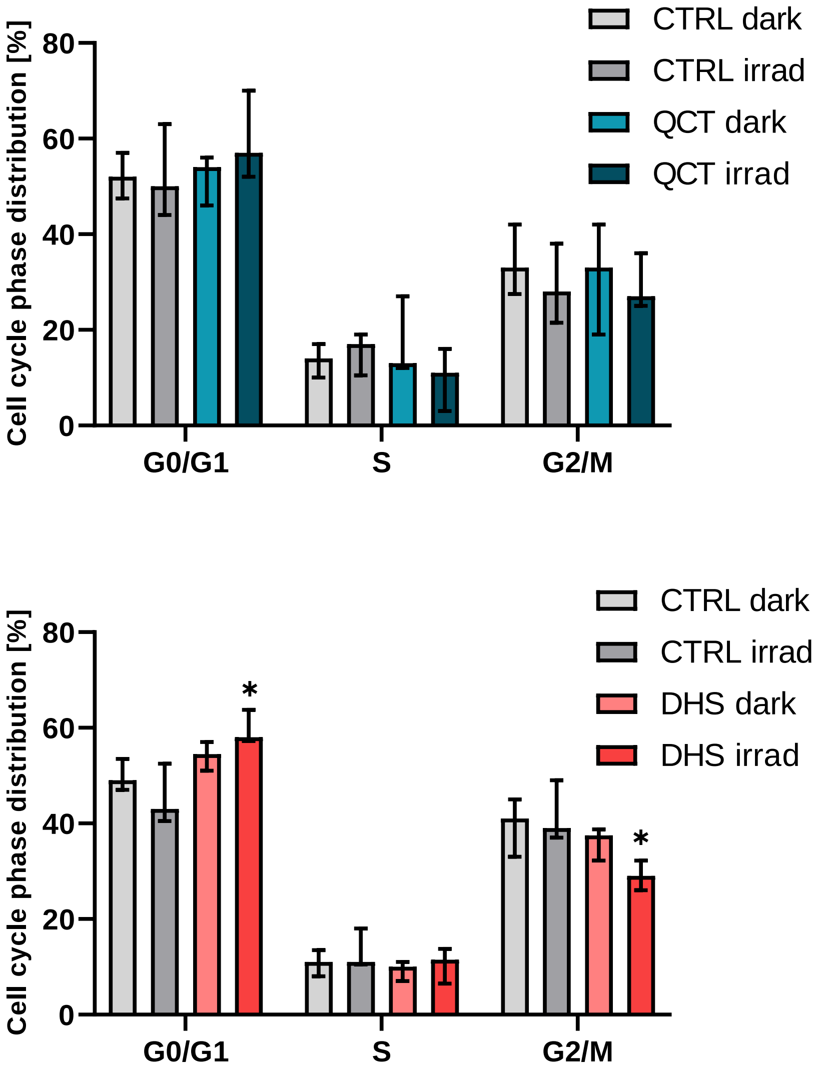

Figure S8. The effect of light irradiation on the cell cycle of human hepatic HepG2 cells exposed to quercetin and 2,3-dehydrosilybin. HepG2 cells were treated with quercetin or 2,3-dehydrosilybin (50 µmol/L) and kept in the dark or irradiated with white light (LED, I = 160 mW/cm^2) for 2 h. Control cells were treated with vehicle (1% DMSO) in cell medium. The cell cycle was measured by flow cytometry after another 22 h in the dark. * $p \leq 0.05$; n ≥ 6

Figure S9. Titration of quercetin and 2,3-dehydrosilybin in sodium chloride solutions. Experiments were performed with 100 μM quercetin (QCT) or 2,3-dehydrosilibyn (DHS) in aqueous sodium chloride solutions (0.16 mol L^{-1}; with 5% DMSO), monitored by UV/vis spectroscopy. NaOH and HCl solutions were gradually added and homogenized before the measurement started. Absorbance values are corrected for volume changes during the titrations.

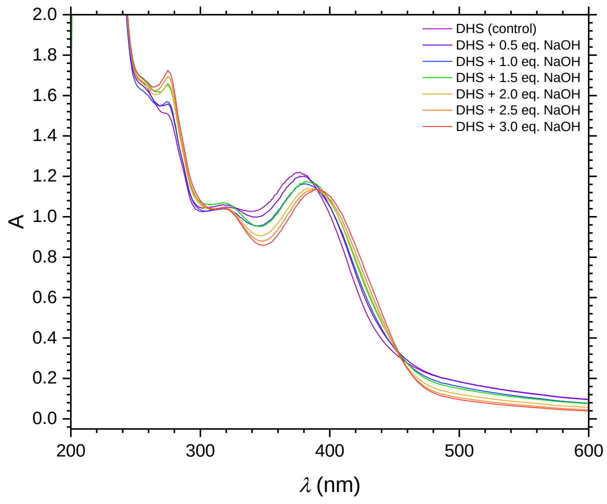

Figure S9A. Titration of DHS (100 μM) with NaOH in a sodium chloride aqueous solution (0.16 mol L^{-1}) with 5% of DMSO.

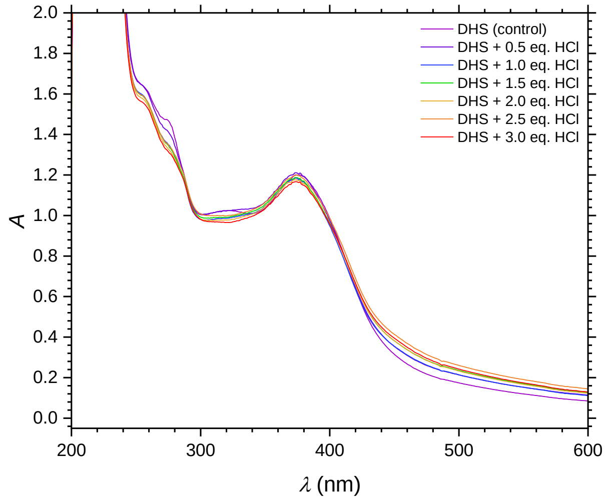

Figure S9B. Titration of DHS (100 μM) by HCl in a sodium chloride aqueous solution (0.16 mol L^{-1}) with 5% of DMSO.

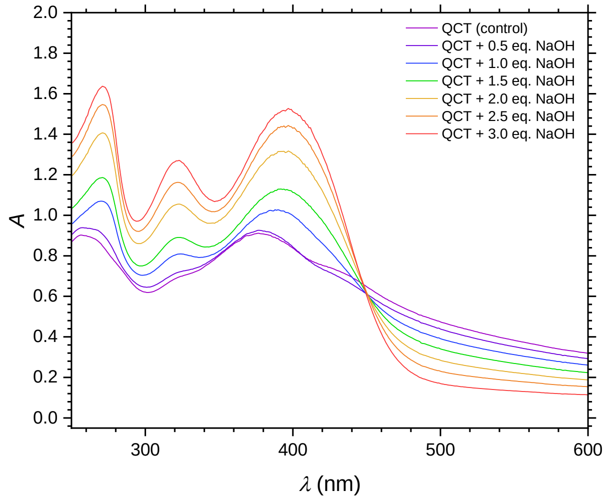

Figure S9C. Titration of QCT (100 μM) by NaOH in a sodium chloride aqueous solution (0.16 mol L^{-1}) with 5% of DMSO.

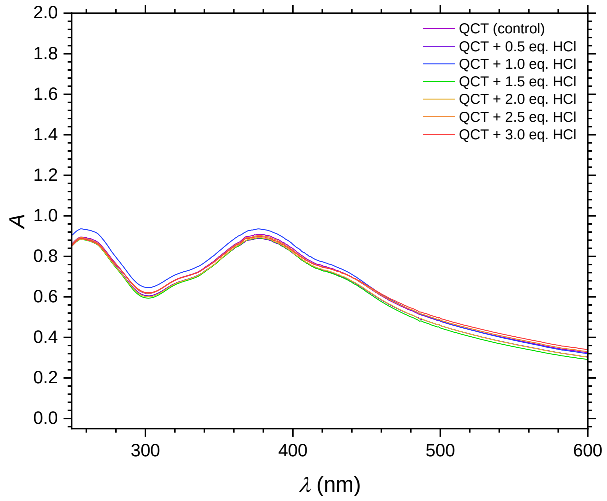

Figure S9D. Titration of QCT (100 μM) by HCl in a sodium chloride aqueous solution (0.16 mol L^{-1}) with 5% of DMSO.

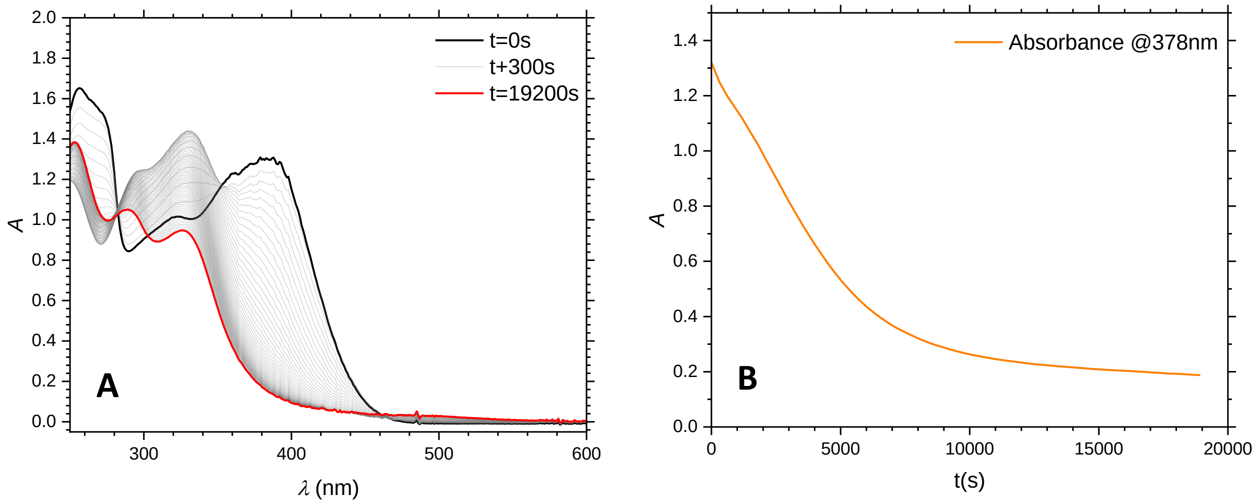

Figure S10. Photodegradation kinetics of quercetin (c = 100 μM), irradiated by LED (λ_{irr} = 400 nm) during exhaustive irradiation. (A) Spectra recorded in a PBS solution (10 mM, pH 7.; with 5% DMSO), taken every 5 min. (B) Absorbance evolution at 378 nm.

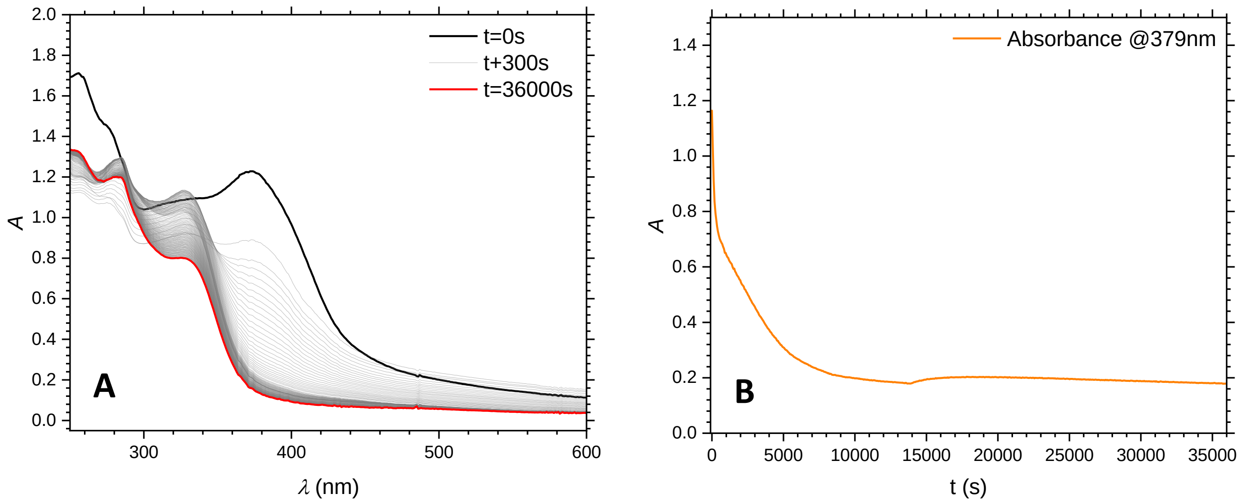

Figure S11. Photodegradation kinetics of 2,3-dehydrosilybin (c = 100 μM)), irradiated by LED (λ_{irr} = 400 nm) during exhaustive irradiation. (A) Spectra recorded in a PBS solution (10 mM, pH 7.4; with 5% DMSO), taken every 5 min. (B) Absorbance evolution over time at 379 nm.
